# (20R)-panaxadiol improves obesity by promoting white fat beigeing

**DOI:** 10.3389/fphar.2023.1071516

**Published:** 2023-02-22

**Authors:** Yuqian Lv, Xiaoyan Lv, Jianshu Feng, Fanghui Cheng, Zhiyi Yu, Fengying Guan, Li Chen

**Affiliations:** ^1^ Department of Pharmacology, School of Basic Medical Sciences, Jilin University, Changchun, China; ^2^ Department of Clinical Laboratory, The Second Clinical Hospital Affiliated to Jilin University, Changchun, China; ^3^ Department of Physiology and Pharmacology, Department of Basic Medicine, Changchun Medical College, Changchun, China

**Keywords:** (20R)-panaxadiol, obesity, beige, β_2_-adrenoceptor, cAMP pathway

## Abstract

**Introduction:** Obesity is an important cause of a range of metabolic diseases. However, the complex mechanisms of obesity and its related diseases make some weight loss methods ineffective or have safety issues. Ginseng, a specialty of Jilin Province in China with both edible and medicinal value, contains mainly ginsenosides and other components. In order to study the anti-obesity effect of ginseng, network pharmacology was used to predict and screen the active ingredients, action targets and signaling pathways of ginseng. We found (20R)-panaxadiol (PD) is a more desirable active ingredient due to its high drug-like properties and high bioavailability. Moreover, it is closely related to cAMP pathway which is more important in metabolism regulation. The corresponding pharmacodynamic targets of PD include ADRB2 (the gene encoding the β_2_-adrenoceptor receptor). Our study aimed to investigate whether Panaxadiol can promote white adipocyte beigeing and increase thermogenesis through modulating the β_2_/cAMP pathway to exert anti-obesity effects.

**Methods:** *In vivo*, we established high-fat feeding obesity model, genotypically obese mice (ob/ob) model, and administered PD (10 mg/kg). PD treatment in ob/ob mice along with β_2_ receptor inhibitor ICI118551. *In vitro*, differentiated mature 3T3-L1 cells were given palmitate (PA) to induce hypertrophy model along with PD (20 μM).

**Results:** The results of this study demonstrated that PD significantly reduced body weight, improved glucose tolerance and lipid levels in high-fat-induced obese mice and ob/ob mice, and also reduced lipid droplet size in PA-treated hypertrophic adipocytes *in vitro*. Molecular biology assays confirmed that cAMP response element binding protein (CREB) phosphorylation was increased after PD administration, and the expression of thermogenesis-related proteins UCP1, PRDM16 and mitochondrial biosynthesis-related proteins PGC-1α, TFAM and NRF1 were increased. Molecular docking results showed a low binding energy between β_2_ receptors and PD, indicating an affinity between the β_2_ receptor and PD. In addition, the β_2_ receptor inhibition, reversed the anti-obesity effect of PD on the body weight, lipid droplets, the expression of thermogenesis-related proteins and CREB phosphorylation in ob/ob mice.

**Discussion:** These results suggest that PD may promote the expression of thermogenic proteins through phosphorylation of CREB *via* β2 receptor activation, and thus exert anti-obesity effects.

## 1 Introduction

Obesity is a pathological state of fat accumulation, which is mainly manifested by the massive accumulation of fat resulting in adipocyte hypertrophy and adipose tissue hyperplasia (Zhang et al., 2017). Obesity has become a public health problem in China and worldwide, and patients suffer psychologically and physiologically problems to some extent. Some studies have shown that the incidence of diabetes is much higher in obese than normal populations, and obesity is a high risk factor for the development of cardiovascular diseases including atherosclerotic and heart failure (Maggio and Pi-Sunyer, 2003; Riobó Serván, 2013). In recent years, the role of effective active ingredients extracted from natural plants, i.e., phytochemicals, has received increasing attention in the prevention and treatment of obesity and obesity-related metabolic diseases (Azhar et al., 2016; Zhao et al., 2017). Several phytochemicals are employed to reduce the process of adipogenesis, carbohydrates absorption in the small intestine, collection of hepatic triglycerides, deposition of adipose tissue, weight loss, and enhances the anti-obesity potential, activity of PPAR-α and PPAR-*γ*-responsive genes (Kumar et al., 2022) (Shehadeh et al., 2021) (González-Castejón and Rodriguez-Casado, 2011). Ginseng is one of the most valuable herbs in traditional Chinese medicine (Yoo et al., 2012; Kim et al., 2017; Li and ji. 2018a; So et al., 2018; Yu et al., 2018), which contains a variety of active ingredients including ginsenosides (Ratan et al., 2021). Ginseng is a plant of high value for both food and medicinal purposes. In China, ginseng has been widely trusted and used as a medicinal herb that can make people strong and healthy (Sabouri-Rad et al., 2017). Recently, the anti-obesity effects of ginseng have been increasingly mentioned. Modern pharmacological studies have repeatedly suggested the weight loss and hypoglycemic effects of ginseng (Li and Ji, 2018b). A study by (Karu et al., 2007) showed that ginseng has some anti-obesity and hypolipidemic effects on high-fat diet-induced obese mice.

However, the bioavailability of each component of ginseng varies, and its specific mechanism of action is unclear for its multi-component and multi-target characteristics. Further study of the material basis, pharmacological efficacy and mechanism of ginseng’s anti-obesity is still needed. Nowadays, the emergence of network analysis has provided new ideas to study the mechanism of action of many herbal medicines such as ginseng. In the previous study, network analysis was used to predict and screen the active ingredients, the corresponding targets and signaling pathways of ginseng. We found (20R)-panaxadiol (PD) is a more desirable active ingredient due to its high drug-like properties and high bioavailability. Moreover, it is closely related to cAMP pathway which is more important in metabolism regulation. The corresponding pharmacodynamic targets of PD include ADRB2 (the gene encoding the *β*_2_-adrenoceptor).

The cAMP pathway has been reported to be associated with thermogenesis in adipose tissue (Liu et al., 2019; Zhang et al., 2021). Adipose tissue can be divided into white and brown adipose. The main function of classical brown fat is to produce heat, while the function of white fat is to store energy, and both are important regulators of energy homeostasis. Recent studies have pointed to the existence of a potential cell type in white adipocytes that can specifically express PRDM16 (PRdomain-containing16), PGC-1α (Peroxisome proliferators-activated receptor-γ coactivator-1) and UCP1 (Uncoupling protein 1), biomarkers for brown-specific genes, known as beige adipocytes (Harms and Seale, 2013). Beigeing, which refers to white fat exhibiting brown fat-like properties after external environmental or pharmacological stimulation, is a phenomenon in which the expression of brown-specific genes in white fat (especially in SWAT). The cAMP signaling pathway plays an important role in the study of promoting beige coloration of white fat. Current studies have demonstrated that by activating *ß*-adrenergic receptors and thus adenylate cyclase, catalyzing the conversion of ATP to cAMP, activates PKA, phosphorylates the cAMP response element binding protein (CREB), and promotes downstream brown-specific genes expression, which increases energy expenditure for the purpose of disease treatment (Fenzl and Kiefer, 2014). In this study, obese mice and PA-treated hypertrophic adipocytes were used to investigate whether 20R-Panaxadiol can promote white adipocyte beigeing and increase thermogenesis through modulating the *β*_2_/cAMP/CREB pathway to exert anti-obesity effects.

## 2 Materials and methods

### 2.1 Materials

The PD used in this study was donated by Professor Yanping Chen (College of Chemistry, Jilin University). Its purity was determined to be more than 98.9% by normalization method of HPLC. Total cholesterol (TC), triglyceride (TG) and total cholesterol (TCHO) diagnostic test kits were purchased from Nanjing Jiancheng Bioengineering Institute (Nanjing, China). Blood glucose test strips were purchased from Roche (Basel, Switzerland). All other reagents were purchased from Beijing Chemical Factory (Beijing, China).

### 2.2 Animals and treatments

The animal study designed in this paper was approved by the Medical Ethics Committee of Jilin University (protocol code 2021156, approval date 20210301). A number of C57BL/6J male mice and ob/ob male mice (3 weeks) were purchased from Beijing HFK Bioscience Co., Ltd. (SCXK 2019-0008) (Beijing, China). The mice were housed in a temperature-controlled animal room with a constant 12 h light/dark cycle.

After 1 week of adaptive feeding, C57BL/6J mice were taken as control group with low fat feed [CONTROL, *n* = 6, Research DIETS (D12450B)], high fat group with high fat feed [*n* = 14, Research DIETS (D12492) ]. After 8 weeks of high-fat feeding, the mice gained 20 percent more weight than the control group were taken as obesity, and the modeling success rate was 85 percent. Then obesity mice randomly were divided into two groups, high fat group (HF, *n* = 6), (20R)-panaxadiol treatment group (PD, *n* = 6). PD was administered by gavage (10 mg/kg) for 5 weeks. CONTROL and HF group were given equivalent amounts of sodium carboxymethyl cellulose.

After 2 weeks of normal feeding, the ob/ob mice gained 20 percent more weight than the control group, were randomly divided into three groups, ob/ob group (OB, *n* = 6), 20R-Panaxadiol group (PD, ig. 10 mg/kg, *n* = 6) and PD+*β*_2_ receptor inhibitor group (ICI118551, ip. 1 mg/kg, *n* = 6). Body weights of mice were measured weekly and treatments were administered by gavage or intraperitoneal injection (equivalent amounts of sodium carboxymethyl cellulose and normal saline were given as controls) from 9:00 to 10:00 a.m. daily for 8 weeks.

HF mice and ob/ob mice were subjected to oral glucose tolerance test (OGTT) at week fourth and week seventh of drug administration, respectively. Mice were anesthetized with isoflurane by inhalation (1.5%) and euthanized by CO_2_ inhalation in the next week. Blood sampling was taken from the medial canthus vein and serum was collected after centrifugation at 3,000 *g* (4°C) for 15 min. Then serum TC, TG, TCHO were determined according to the manufacturer’s instructions. Samples of subcutaneous adipose tissue (SWAT) and interscapular brown adipose tissue (BAT) were extracted for weighing.

### 2.3 OGTT

For the OGTT, mice were fasted for 12 h and then orally gavaged with glucose dissolved in water at 2 g/kg body weight. Ten microliters of blood was obtained from the tail tip, and the concentration of glucose was measured at 0, 30, 60, 90, and 120 min.

### 2.4 Hematoxylin and eosin (HE) and immunofluorescence (IF) stainings

The adipose tissue was quickly removed from the mice, washed with normal saline, dried and then fixed with 10% formalin. After rapid removal of the adipose tissue, it was placed in 4% paraformaldehyde. It was then dehydrated in an ascending series of ethanol, and equilibrated with xylene, followed by embedding in paraffin and sectioning into 5–10 µm slices. Then, the samples were dewaxed with xylene and a descending series of ethanol. Continued sections were stained with both Mayer’s hematoxylin and eosin (HE).

Dried paraffin sections were dewaxed and hydrated and then closed with 1% BSA for 30 min, the closure solution was blotted dry on blotting paper. Each section was incubated with UCP1 antibody (abcam1:100) overnight at 4°C in a wet box, and washed 3 times with PBS. After aspirating the residual liquid, the sections were incubated with fluorescent secondary antibody (FITC1:100) for 1 h at room temperature in a wet box protected from light, then rinse 3 times with PBS, the excess liquid was aspirated. A small amount of anti-fluorescence quencher containing DAPI dropwise was added, the sample was covered with a coverslip, and stored in a wet box protected from light for observation under a fluorescence microscope for photographs.

### 2.5 Cell culture

3T3-L1 preadipocytes (ATTC, United States) were cultured in Dulbecco’s modified Eagle’s medium (DMEM) and 10% bovine calf serum (Gibco, United States) in an atmosphere of 5% CO_2_ at 37°C. For differentiation studies, 3T3-L1 cells were cultured in DMEM containing 10% FBS,^1^ μMOL dexamethasone (Sigma, United States), 0.5 mMOL IBMX (Sigma, United States) and 5 μg/mL insulin for 2 days and then replaced with DMEM culture medium without 10% FBS and 5 μg/mL insulin for 2 days. Then replaced with DMEM containing 10% FBS. DMEM cell culture medium containing 10% FBS was changed every 2 days until the 8–10th^ ^day.

### 2.6 Oil red O staining

Differentiated mature 3T3-L1 cells were given PA and PD for 24 h. The cells were gently rinsed with PBS before staining, followed by fixation with 4% paraformaldehyde for 1 h. Then cells were washed with PBS, and each well was stained with 2 mL of freshly configured Oil Red O working solution for 1–2 h. The staining solution was discarded, and 60% isopropanol was rinsed quickly once, followed by rinsing with ultrapure water three times, and observed under the microscope for photographs.

### 2.7 Immunoblot analysis

Proteins were extracted from cell lysates following the manufacturer’s protocols (Beyotime, China). Protein concentration was quantified using the BCA protein assay kit (Thermo Fisher Scientific, United States) and 30 μg protein was separated in a 12% SDS polyacrylamide gel and electro transferred onto polyvinylidene difluoride (PVDF) membranes (Bio-Rad, United States). Membranes were blocked with 5% (w/v) BSA for 2 h at room temperature and then incubated with primary antibodies with light shaking overnight at 4°C. Primary antibodies against UCP1, PGC-1α, TFAM, NRF1, CREB, P-CREB and GAPDH (abcam) were diluted to a ratio of 1:1,000 in TBST buffer. The membranes were washed 3 times for 5 min each with 10 mL of TBST [10 mM Tris-HCl, 150 mM NaCl and 0.1% (v/v) Tween-20] and then incubated with secondary antibody at room temperature for 2 h. Secondary antibodies goat anti-rabbit or goat anti-mouse (Proteintech, United States) were diluted to a ratio of 1:5,000 in TBST buffer. The membrane was incubated in Western ECL substrate (Thermo fisher or Proteintech, United States) and exposed to Tanon imager, using ImageJ software for image analyses.

### 2.8 Statistical analysis

All data are expressed as mean ± standard error of the mean. Statistical significance of differences was analyzed by one-way ANOVA with Dunnett test using GraphPad Prism8. *p* < 0.05 was considered to indicate a statistically significant difference.

## 3 Result

### 3.1 Anti-obesity effect of PD on HF mice

**FIGURE 1:**
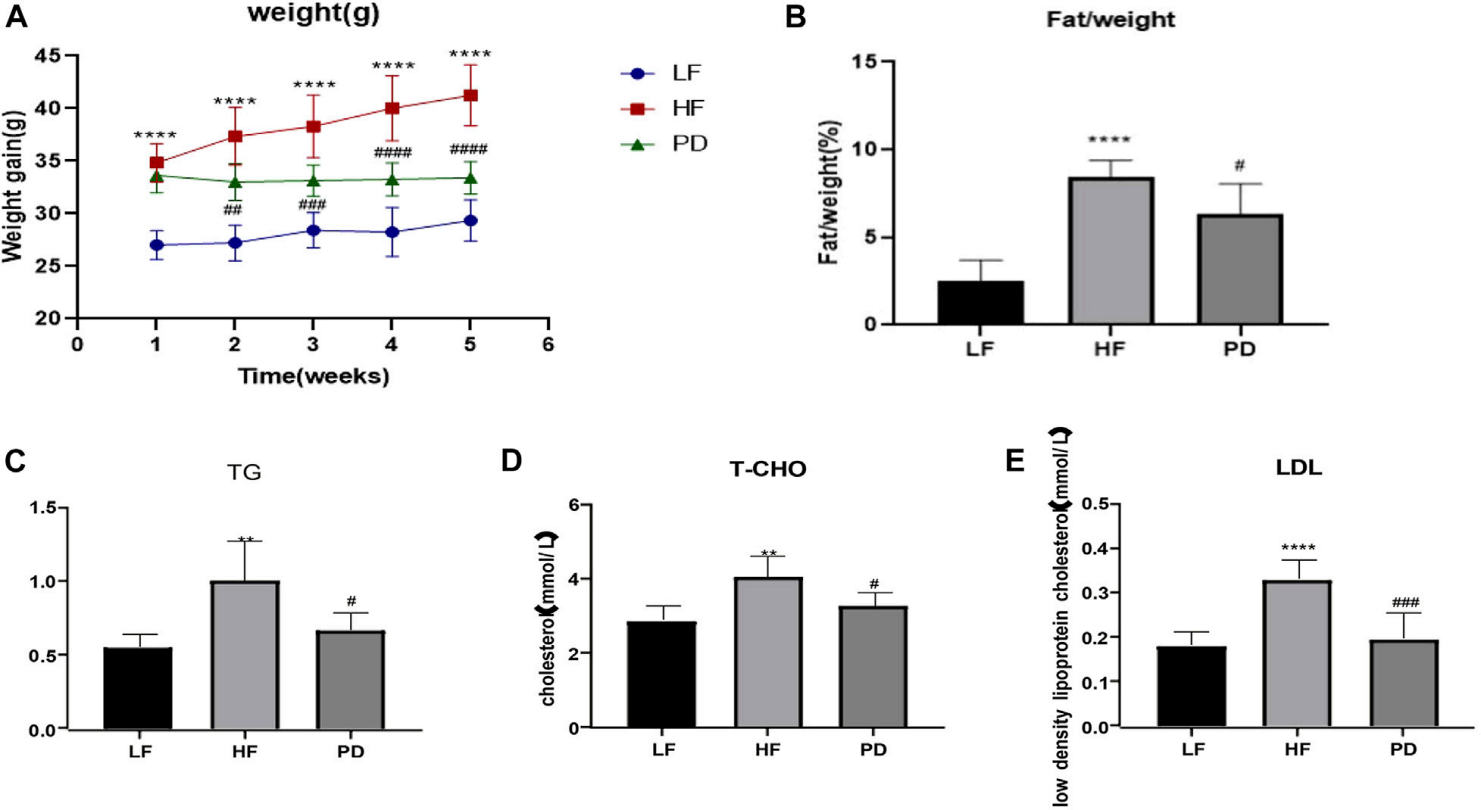
Anti-obesity effect of PD on HF mice **(A, B)** The changes of body weight and fat/weight (g) during the experimental course; **(C–E)** Serum lipid profile including circulating TG, TCHO and LDL levels; ^**^ *p* < 0.01, ^****^ *p* < 0.0001, compared with the LF group. ^#^ *p* < 0.05, ^##^ *p* < 0.01, ^###^ *p* < 0.001, ^####^ *p* < 0.0001, compared with the HF group. *n* = 6.

As shown in Figures 1A,B, after 5 weeks of PD administration, the body weight of mice in PD group was significantly lower than that of the model group, and the body fat ratio was significantly reduced. It is suggested that PD can reduce the body weight and body fat ratio of obese mice induced by high-fat diet. Serum TG, T-CHO and LDL (Figures 1C–E) were significantly reduced after 5 weeks of PD treatment, suggesting that PD can effectively improve the lipid levels in high-fat diet induced obese mice.

### 3.2 Effects of PD on glucose tolerance, fasting glucose and subcutaneous inguinal white fat morphology in HF mice

**FIGURE 2:**
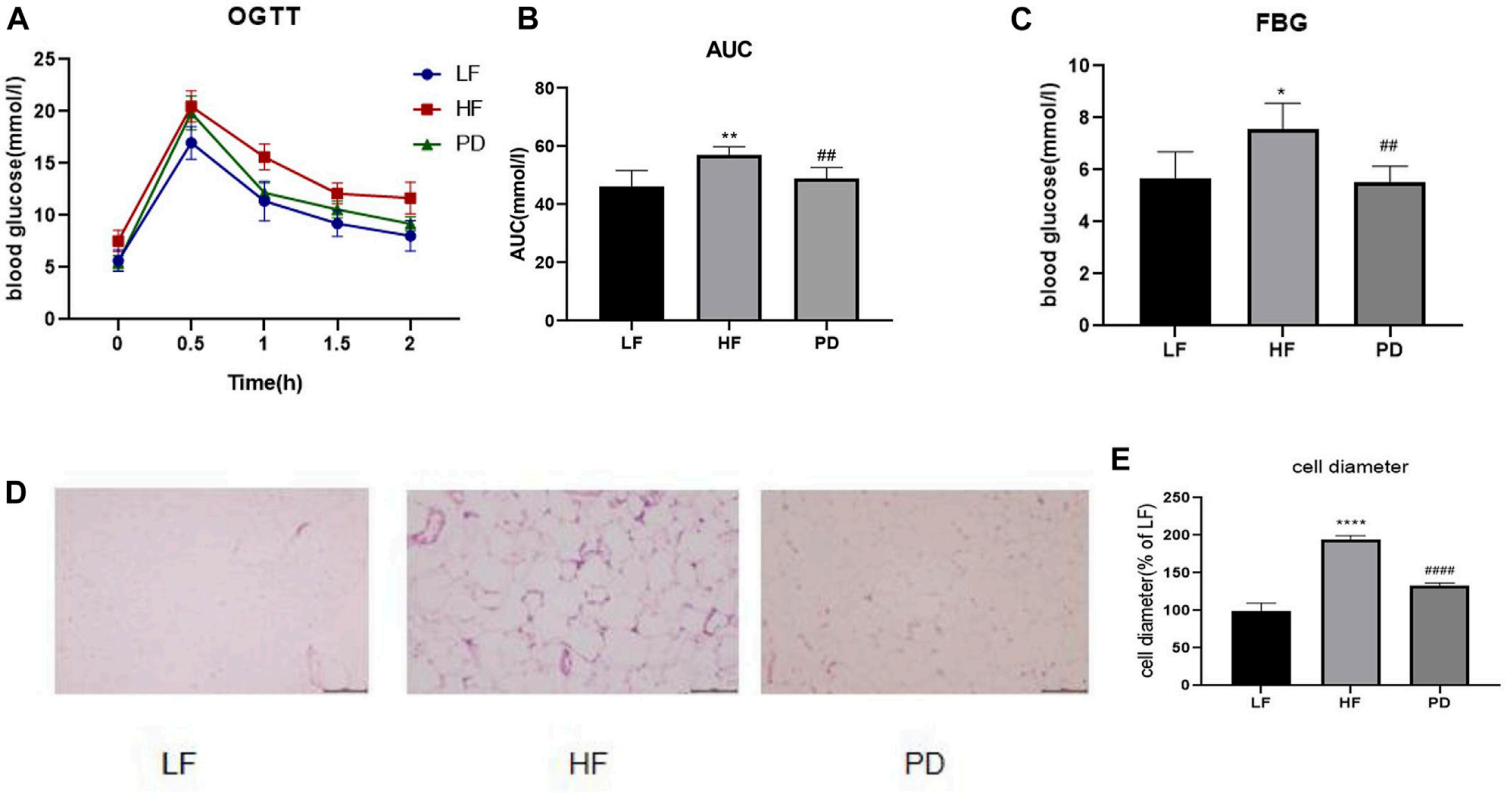
Effects of PD on glucose tolerance, fasting glucose and subcutaneous inguinal white fat morphology in HF mice **(A)** OGTT after 4 weeks of PD treatment; **(B)** AUC of each group was calculated during the oral glucose tolerance test; **(C)** Fasting blood glucose level in each group; **(D)** PD alleviated morphology changes of SWAT in HF mice; **(E)** Cell diameter changes in each group; ^*^ *p* < 0.05, ^**^ *p* < 0.01, ^****^ *p* < 0.0001, compared with the LF group. ^##^ *p* < 0.01, ^####^ *p* < 0.0001 compared with the HF group. *n* = 5.

Obesity is often accompanied by varying degrees of impairment in glucose tolerance, so we used OGTT to test the effect of PD on glucose tolerance. As shown in the Figures 2A–C, after PD treatment, the AUC (Area Under Curve) of blood glucose in the PD group was lower than that of the model group, and its recovery level was significantly faster than that of the model group, and fasting blood glucose was significantly lower. The above results suggest that the elevated fasting glucose and impaired glucose tolerance in obese mice induced by high-fat diet were improved to some extent after the administration of PD. The results of HE staining of adipose tissue (Figures 2D, E) showed that after the administration of PD treatment, the volume and diameter of adipocytes were significantly reduced and the intercellular spaces were dense. It is suggested that PD has an ameliorative effect on the morphology of subcutaneous inguinal white adipose tissue in high-fat diet induced obese mice.

### 3.3 Effect of PD on thermogenic and cAMP related pathway proteins in adipose tissue of HF mice

**FIGURE 3:**
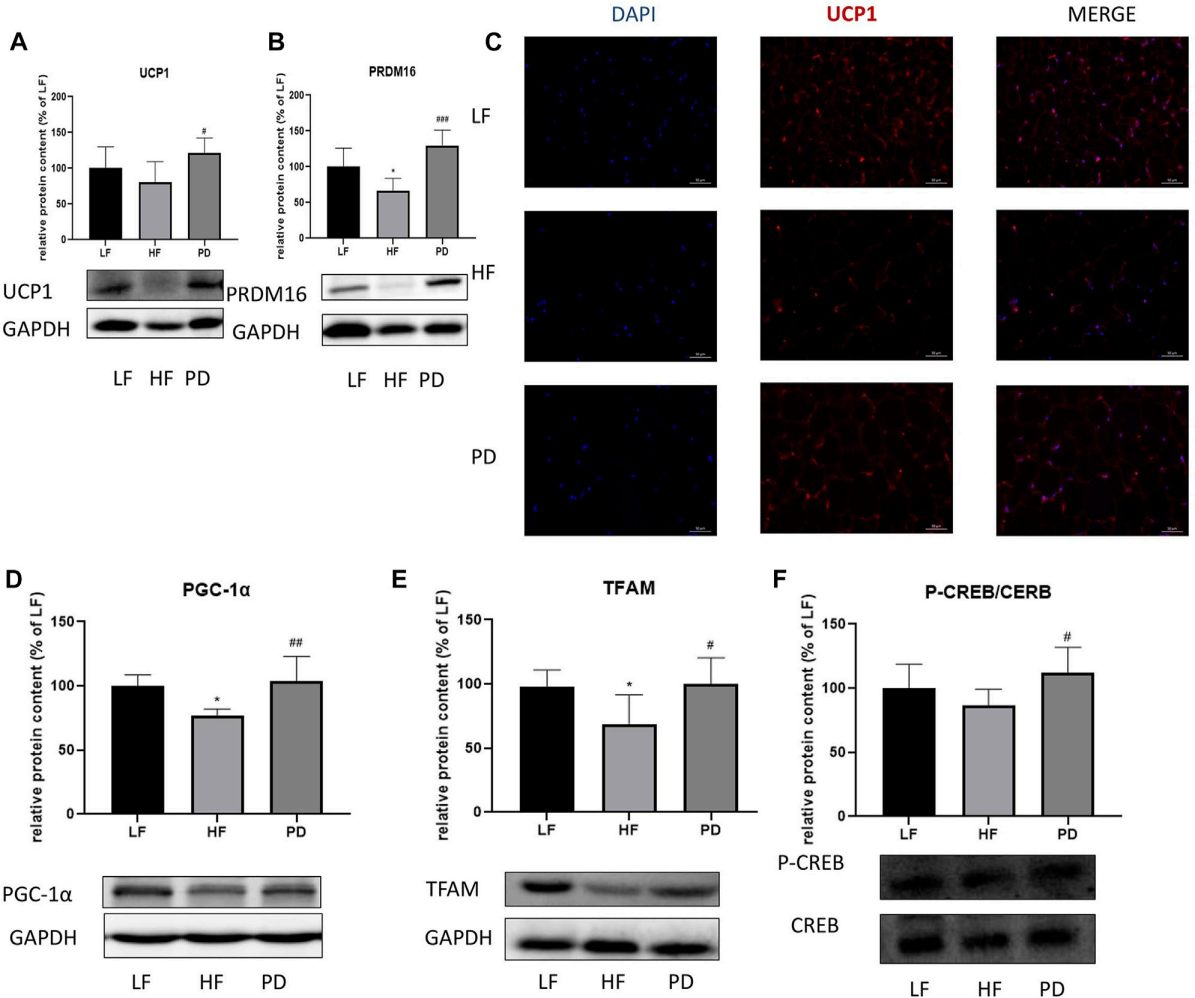
Effect of PD on thermogenic and cAMP related pathway proteins in adipose tissue of HF mice PD increased the expression of thermogenesis-related proteins in the HF group of mice. **(A, C)** UCP1 protein expression; **(B)** PRDM16 protein expression; PD increases the expression of mitochondrial biosynthesis-related proteins PGC-1α **(D)** and TFAM **(E)**; **(F)** P-CREB content increased significantly after the administration of PD treatment; *p* < 0.05, compared with the LF group. ^#^ *p* < 0.05, ^##^ *p* < 0.01, ^###^ *p* < 0.001 compared with the HF group. *n* = 6.

To investigate the anti-obesity mechanism of PD, we examined the expression of thermogenic proteins PRDM16 and UCP1, mitochondrial biosynthesis-related protein PGC-1α, TFAM and cAMP pathway related protein P-CREB in adipose tissue after (20R)-panaxadiol administration. The results are shown in the Figures 3A, B, the expression of thermogenic proteins PRDM16 and UCP1 in PD treatment mice were significantly increased in immunofluorescence (Red fluorescence represents the expression of UCP1, Figure 3C). The results were the same as the western blotting results. Mitochondrial biosynthesis-related proteins PGC-1α, TFAM expression were also significantly increased (Figures 3D, E). In order to initially investigate the causes of the changes in adipose tissue thermogenesis and mitochondrial biosynthesis, we used Western blotting to detect the phosphorylation of the cAMP pathway related protein CREB, and as shown in the Figure 3F, P-CREB content increased significantly after the administration of PD. It is suggested that PD can promote thermogenesis and mitochondrial biosynthesis, and this effect may be related to the cAMP/CREB pathway.

### 3.4 *In vitro* experiments to study the ameliorative effect of PD on hypertrophic adipocytes and its association with cAMP related pathway proteins

**FIGURE 4:**
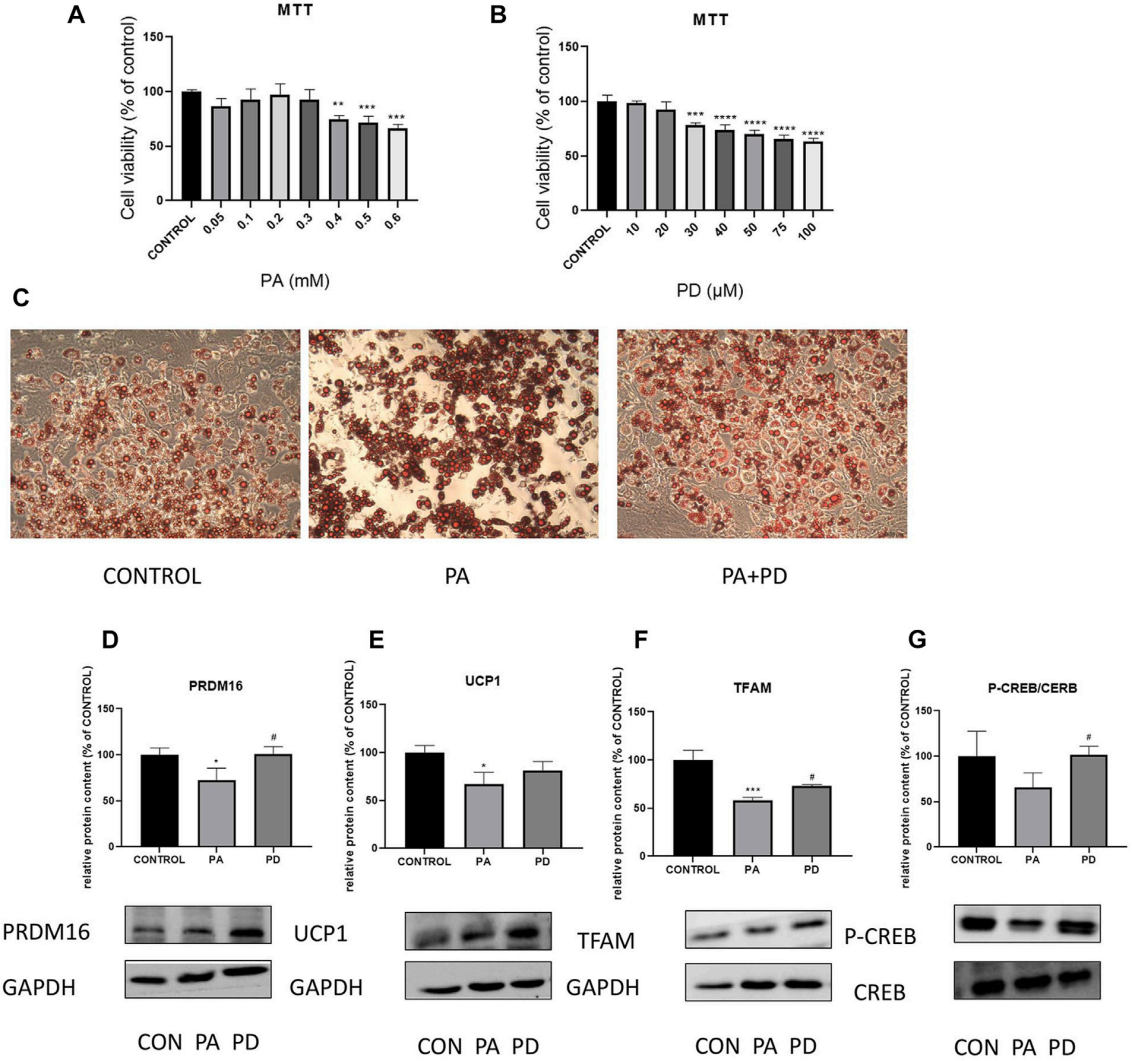
*In vitro* experiments to study the ameliorative effect of PD on hypertrophic adipocytes and its association with cAMP related pathway proteins MTT assay to screen PA-modeling concentration **(A)** and PD administration concentration **(B)**; **(C)** Oil Red O staining; **(D)** PRDM16 protein expression; **(E)** UCP1 protein expression; PD increases the expression of mitochondrial biosynthesis-related proteins TFAM **(F)**; **(G)** P-CREB content increased significantly after the administration of PD treatment; ^*^ *p* < 0.05, ^**^ *p* < 0.01, ^***^ *p* < 0.001, ^****^ *p* < 0.0001, compared with the CONTROL group. ^#^ *p* < 0.05 compared with the PA group. *n* = 3.

*In vitro* experiments were performed using MTT assay to screen PA-modeling concentration of 0.3 mMOL and PD administration concentration of 20 μMOL. The results are shown in the Figure 4C, the lipid droplet volume of PA-stimulated adipocytes was increased compared with the control group. After giving PA stimulation along with PD protection, the lipid droplet volume was reduced compared with the PA group. To investigate the mechanism of action of 20R-Panaxadiol in *vitro* experiments, we examined the expression of the thermogenic proteins PRDM16 and UCP1, the mitochondrial biosynthesis-related protein TFAM and the cAMP pathway related protein P-CREB after administration of PD to hypertrophic adipocytes. As shown in the Figures 4D–G, PRDM16, TFAM and P-CREB expression were significantly increased after PD administration. It is suggested that at the cellular level, (20R)-panaxadiol also has the same effect of promoting thermogenesis and increasing mitochondrial biosynthesis in hypertrophic white adipocytes, and this effect may be through the cAMP/CREB pathway.

### 3.5 Anti-obesity effect of PD *via β*_2_ receptor in ob/ob mice

**FIGURE 5:**
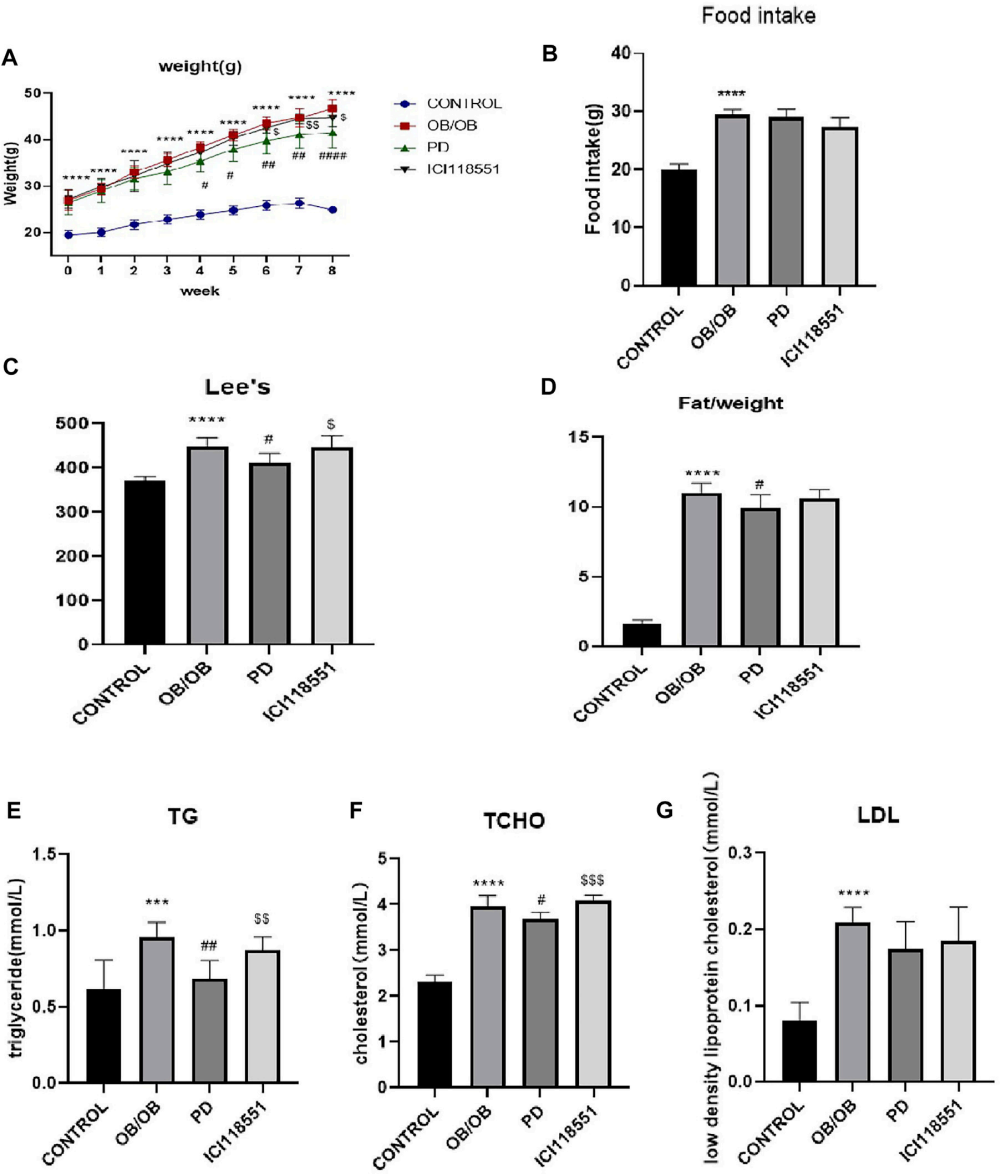
Anti-obesity effect of PD *via β*_2_ receptor in ob/ob mice **(A–D)** the changes of body weight, food take, Lee’s index and fat/weight (g) during the experimental course; **(E–G)** Serum lipid profile including circulating TG, TCHO and LDL levels; ^***^ *p* < 0.001, ^****^ *p* < 0.0001, compared with the CONTROL group. ^#^ *p* < 0.05, ^##^ *p* < 0.01, ^####^ *p* < 0.0001compared with the OB group. ^$^ *p* < 0.05, ^$$^ *p* < 0.01, ^$$$^ *p* < 0.001, compared with the PD group. *n* = 6.

To investigate whether the weight loss effect of 20R-Panaxadiol acts through the *β*_2_ receptor, we administered PD on genotypically obese mice ob/ob mice, and the *β*_2_ receptor was inhibited by *β*_2_ receptor inhibitor (ICI118551) while PD was administered. As can be seen from Figure 5A, at week four of administration, the weight gain of the PD administered group slowed down significantly and a significant difference was observed compared to the weight of the model group. At the sixth week of administration, there was a significant difference between the body weight of the *β*_2_ receptor inhibitor group and that of PD treatment group. There was no significant difference in the food intake of ob/ob mice in each group (Figure 5B). As shown in Figure 5C, the lee’s index increased significantly in ob/ob mice and decreased significantly after PD treatment, while it increased significantly in the ICI118551 group compared with PD group. Figure 5D shows that the body fat ratio of ob/ob mice increased significantly compared with the control group and decreased significantly after PD treatment for 8 weeks. It was suggested that PD reduced body weight, body fat ratio and lee’s index in ob/ob mice, and that *β*_2_ receptor inhibitors reversed this alteration. After PD administration, serum TG and T-CHO were significantly reduced in ob/ob mice, and LDL was also reduced but without significant difference. Compared with the PD-treated group, mice in the ICI118551 group showed significant increases in TG and T-CHO (Figures 5E–G). It was suggested that PD treatment could effectively improve lipid levels in ob/ob mice, and this effect would be diminished after *β*_2_ receptor inhibition.

### 3.6 Effects of PD on glucose tolerance and subcutaneous inguinal white fat morphology in ob/ob mice *via β*_2_ receptors

**FIGURE 6:**
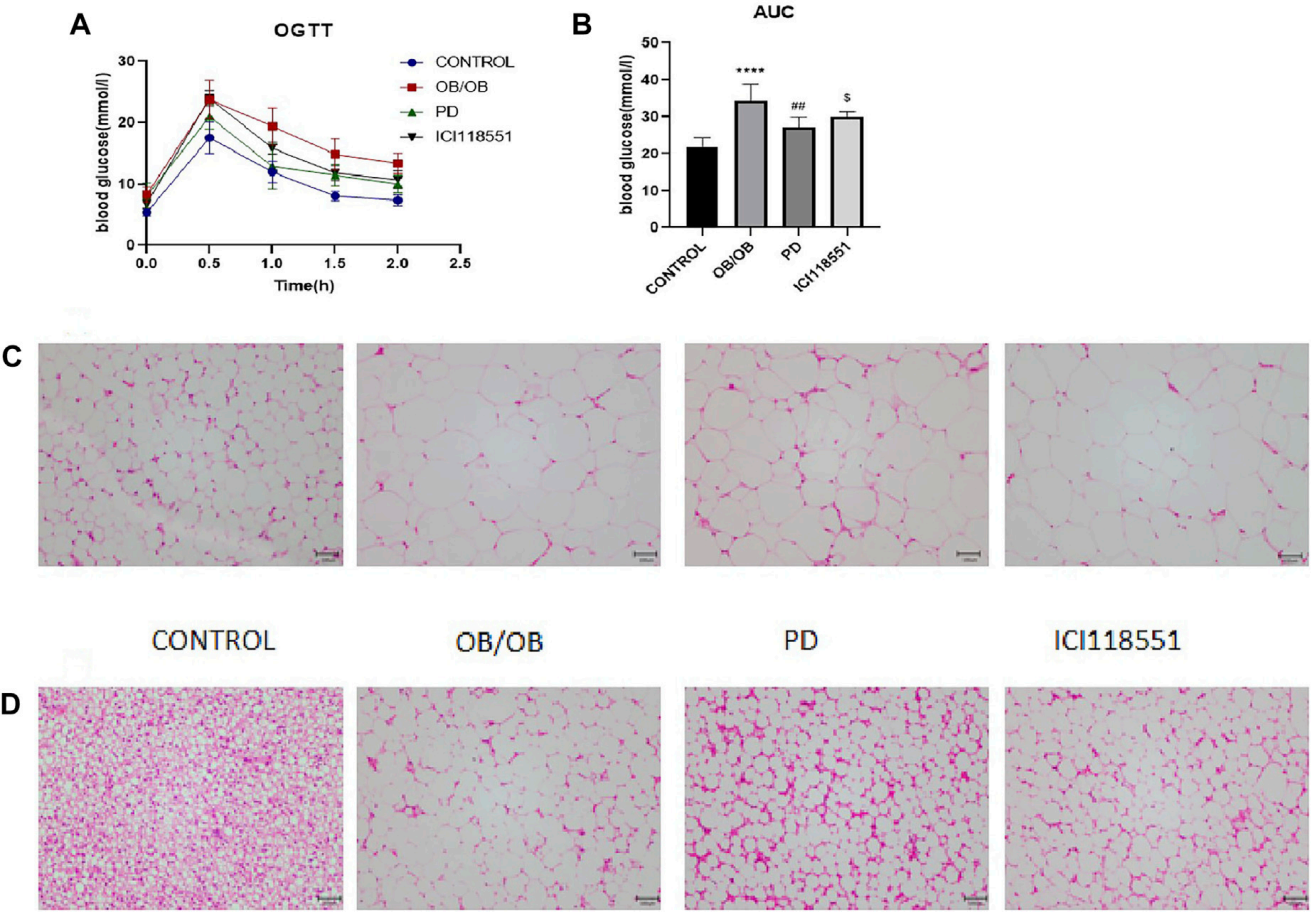
Effects of PD on glucose tolerance and subcutaneous inguinal white fat morphology in ob/ob mice *via β*_2_ receptors **(A)** OGTT after 7 weeks of PD treatment; **(B)** AUC of each group was calculated during the oral glucose tolerance test; PD alleviated morphology changes of **(C)** SWAT and **(D)** BAT in HF mice; ^****^ *p* < 0.0001, compared with the CONTROL group. ^##^ *p* < 0.01, compared with the ob/ob group. ^$^ *p* < 0.05 compared with the PD group. *n* = 5.

To investigate the effect of PD on glucose tolerance in genotypically obese mice, we examined the changes in glucose tolerance in mice. As can be seen from the Figures 6A, B, after PD treatment, the AUC of blood glucose in PD treatment group was lower than that of the model group, and its rate of return to normal level was significantly higher. The blood glucose of mice in the ICI118551 group was higher than that in the PD administration group. The above results suggest that glucose tolerance appears significantly impaired in ob/ob mice and is improved to some extent in ob/ob mice after the administration of PD, which may be diminished by *β*_2_ receptor inhibitors. HE results (Figures 6C, D) showed that after PD treatment, subcutaneous inguinal white adipocytes were significantly reduced in volume and had dense cell gaps. After administration of the *β*_2_ receptor inhibitor, the fat volume was larger compared to the PD administered group. The brown adipose fat cells in the back of PD treatment group were also significantly reduced in size, small multi-chambered lipid droplets started to appear, and the cell interstices were dense. Dorsal fat multicompartmentality was reduced after administration of ICI118551. It was suggested that PD improved adipose histomorphology in genotypically obese mice and could be reversed to some extent by *β*_2_ receptor inhibitors.

### 3.7 Effect of PD on adipose tissue thermogenic proteins and cAMP pathway related protein in ob/ob mice *via β*_2_ receptors

**FIGURE 7:**
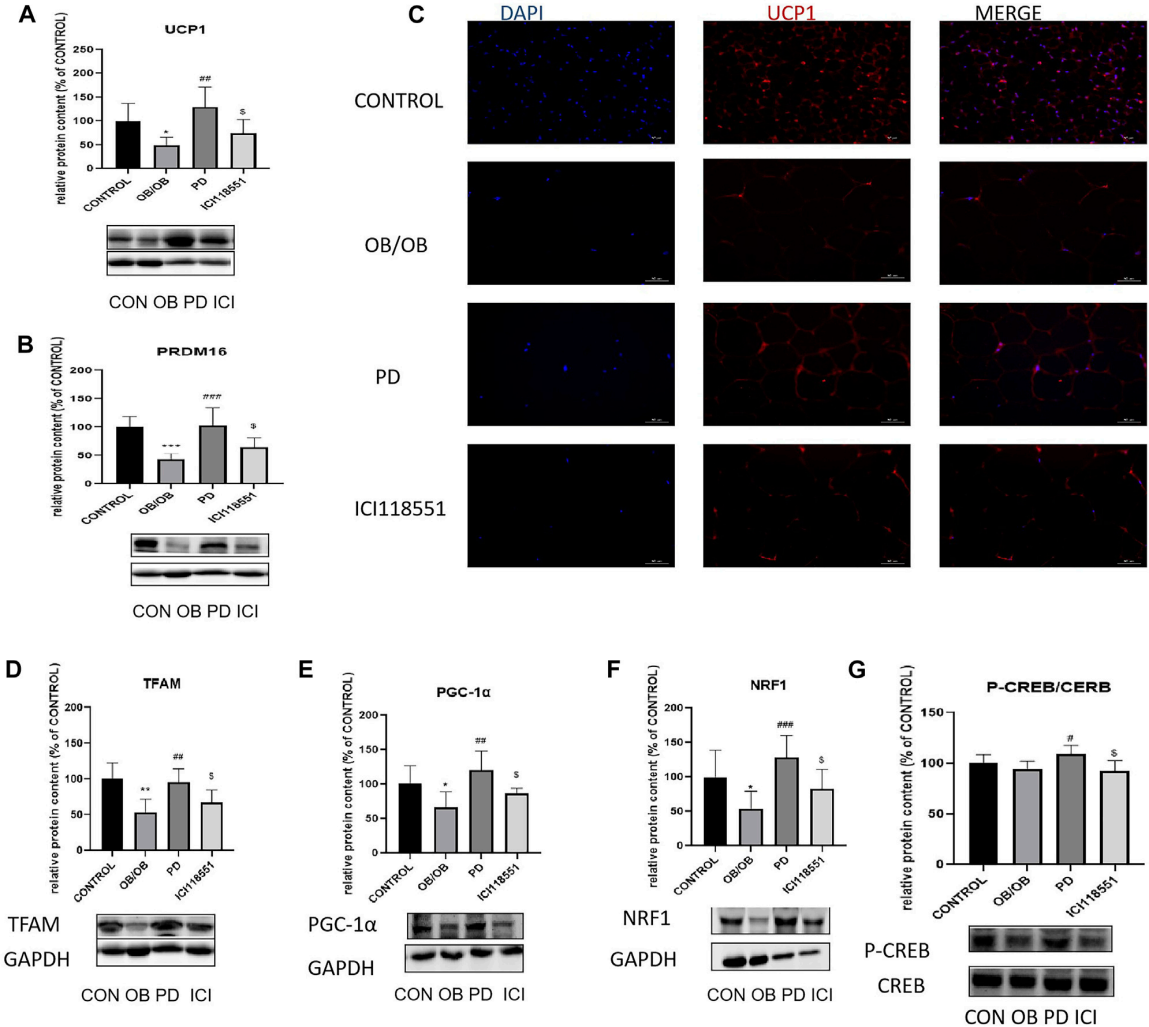
Effect of PD on adipose tissue thermogenic and cAMP related pathway proteins in ob/ob mice *via β*_2_ receptors PD increased the expression of thermogenesis-related proteins in the OB group of mice. **(A, C)** UCP1 protein expression; **(B)** PRDM16 protein expression; PD increases the expression of mitochondrial biosynthesis-related proteins TFAM **(D)**, PGC-1α **(E)** and NRF1 **(F)**; **(G)** P-CREB content increased significantly after the administration of PD treatment; ^*^ *p* < 0.05, ^**^ *p* < 0.01, ^***^ *p* < 0.001, compared with the LF group. ^#^ *p* < 0.05, ^##^ *p* < 0.01, ^###^ *p* < 0.001, compared with the OB group. ^$^ *p* < 0.05 compared with the PD group. *n* = 6.

To investigate the changes in adipose tissue thermogenesis in ob/ob mice, we used Western blotting to detect the expression of thermogenesis-related proteins PRDM16, UCP1 and mitochondrial biosynthesis-related proteins PGC-1α, TFAM, NRF1 and cAMP pathway related protein CREB in subcutaneous SWAT. The results are shown in the Figures 7A,B, the expression of heat-producing proteins PRDM16 and UCP1 significantly increased after PD treatment (Red fluorescence represents the expression of UCP1, Figure 7C). Immunofluorescence results were the same as the Western blotting results. Mitochondrial biosynthesis-related proteins PGC-1α, TFAM, NRF1 expression were also significantly increased (Figures 7D–F). Thermogenic and mitochondrial biosynthesis-related proteins were significantly decreased in adipose tissue of mice given *β*_2_ receptor inhibitors. To initially investigate the causes of adipose tissue thermogenesis and changes in mitochondrial biosynthesis, we used Western blotting to detect the phosphorylation of the cAMP pathway related protein CREB. As shown in the Figure 7G, P-CREB levels increased significantly after PD administration and decreased significantly after *β*_2_ receptor inhibitor administration. It is suggested that the role of (20R)-panaxadiol in promoting thermogenesis and mitochondrial biosynthesis may be through the cAMP/CREB pathway, and this pathway may be mediated by *β*_2_ receptors.

## 4 Discussion

Clinical findings suggest that ginseng may improve cardiovascular function, immune function, and diabetes-related diseases (Wu et al., 2014). showed that ginsenoside Rb1 regulates appetite and achieves energy balance by modulating the expression of inflammatory factors *in vivo* and restoring the anorexigenic effects of leptin and leptin p-STAT3 signaling in the hypothalamus in mice fed a high-fat diet. *In vitro* studies have shown that ginsenosides can inhibit triglyceride synthesis, cholesterol production (Lee et al., 2015), gluconeogenesis (Quan et al., 2012) by activating AMPK and inhibiting fatty acid synthase (FAS) and 3-hydroxy-3-methyl-glutaryl coenzyme A reductase (HMGCR). Ginseng reduced adipocyte size and fat storage in high-fat diet-induced obese mice and rats (Lee et al., 2010). In conclusion, it can be seen that ginseng has certain anti-obesity effects. However, it has multi-component, multi-target and complex pathways, so there is still a long way to go to find the exact and efficient active ingredients and to elucidate their mechanisms of action. Our study found that: 1. (20R)-panaxadiol has a weight loss effect on both high-fat diet-induced and genotypically obese mice. 2. (20R)-panaxadiol can regulate the cAMP pathway, phosphorylate CREB, promote white adipocyte beige, and play a role in promoting thermogenesis and anti-obesity. 3. The anti-obesity effect of (20R)-panaxadiol may be due to the excitement of *β*_2_ receptor.

Activation of the cAMP pathway can phosphorylate the cAMP effector element binding protein (CREB), which in turn promotes the expression of downstream thermogenic genes such as UCP1 (Reverte-Salisa et al., 2019). Recent studies have shown that beige adipocytes present in white adipocytes can also specifically express brown-specific genes such as UCP1 induced by specific external conditions to promote thermogenesis in the organism (Wu et al., 2012). This process of conversion of white fat to beige fat is called white fat beigeing. Based on the above predictions of network analysisand the support of related literature, this project was carried out to verify the effective components and mechanism of action of ginseng against obesity by conducting *in vitro* and *in vivo* experiments. Our results suggested that PD might promote the expression of thermogenic proteins in adipocytes through the cAMP/CREB pathway and thus exert anti-obesity effects.

The well-recognized upstream target of the classical signaling pathway of cAMP is the *ß* receptor, and the *ß* receptor signaling pathway plays an important role in promoting thermogenesis and beigeing (Ohyama et al., 2016; Lim et al., 2019), of which the *β*_3_ receptor has been studied mostly. However, it has been found that the effects of *β*_3_ receptor agonists given in some obese populations are not significant (Redman et al., 2007; Carey AL 2013 January; 56 (1)). Moreover, it has been reported that *β*_3_ receptor expressed in obese animals and humans with impaired functional activity. For example, a variant allele of the human *β*_3_ receptor gene (64 Trp/Arg) has been associated with inhibition of *β*_3_ receptor signaling (Pietri-Rouxel et al., 1997; Kimura et al., 2000) and increased body mass index (BMI), then obesity was promoted (Sipilainen et al., 1997; Mitchell et al., 1998). Recently, it has been shown that *β*_2_ receptor activation in adipocytes also promotes the activation of the cAMP/PKA pathway and exerts some anti-obesity effects. Li et al. (Li et al., 2012) showed a significant reduction in adiposity in pigs after administration of *β*_2_ receptor agonists, and (Ohyama et al., 2016) showed that a subtype of capsaicin could also exert some anti-obesity effects through *β*_2_ receptors. This also suggests that the role of *β*_2_ receptors in the *ß* receptor family in our anti-obesity effect needs to be looked at. After the PPI network analysis of target protein interaction, the degree of target ADRB2 (i.e., *β*_2_ receptor coding gene) was 20, which was much higher than the average degree of 13.4. It indicated that ADRB2 may be the main pharmacological target of ginseng. In addition, the active ingredient corresponding to the target ADRB2 is also (20R)-panaxadiol. Our study subsequently used *β*_2_ receptor inhibitor to intervene in (20R)-panaxadiol treatment of ob/ob obese mice. It was found that inhibition of *β*_2_ receptor reversed the effect on weight loss and upregulation cAMP thermogenesis signal pathway of 20R-Panaxadiol. Based on the above theoretical calculation results and experimental validation, it can be concluded that (20R)-panaxadiol can regulate CREB phosphorylation through activating *β*_2_ receptor and promote the improvement of body thermogenesis and the corresponding mitochondrial biosynthesis, thus achieving the anti-obesity effect.

In summary, (20R)-panaxadiol, as the active component of ginseng, can exert anti-obesity effects by increasing non-tremor thermogenesis and increasing energy expenditure in the body. The mechanism of action may be to act on *β*_2_ receptors and promote phosphorylation of cAMP response element binding protein CREB, which in turn upregulates the expression of downstream thermogenic proteins such as UCP1 and PRDM16. Actually, Weight loss depends on energy expenditure of multiple organs, such as the muscle, the liver, and the adipose tissue. So we think PD may improve obesity by promoting white fat beigeing in part. In subsequent studies, we will take the studies to reveal the energy expenditure in other organs such as skeletal muscle, and on the alleviation of NAFLD. A comprehensive discussion of the effects of PD on obesity related metabolic diseases would be conducted.

## Data Availability

The datasets used and/or analysed during this study are available from the corresponding author on reasonable request.

## Footnotes

1
https://www.jianguoyun.com/p/DULpGzcQgoOICxjQyeIEIAA.

## References

[B1] Azhar Y. Parmar A. Miller C. N. Samuels J. S. Rayalam S. (2016). “Phytochemicals as novel agents for the induction of browning in white adipose tissue.” Nutr. Metab. (Lond) 13, 89. 10.1186/s12986-016-0150-6 27980598 PMC5135798

[B2] Carey A. L. F. M. Van every B. Bertovic D. eikelis N. lambert G. W. kalff V. (2013). Ephedrine activates Brown adipose tissue in lean but not obese humans, Clinical Trial 56, 147-55. 10.1007/s00125-012-2748-1 23064293

[B3] Fenzl A. Kiefer F. W. (2014). “Brown adipose tissue and thermogenesis.” Horm. Mol. Biol. Clin. Investig. 19 (1), 25–37. 10.1515/hmbci-2014-0022 25390014

[B4] González-Castejón M. Rodriguez-Casado A. (2011). Dietary phytochemicals and their potential effects on obesity: A review. Pharmacol. Res. 64 (5), 438–455. 10.1016/j.phrs.2011.07.004 21798349

[B5] Harms M. Seale P. (2013). Brown and beige fat: Development, function and therapeutic potential. Nat. Med. 19 (10), 1252–1263. 10.1038/nm.3361 24100998

[B6] Karu N. Reifen R. Kerem Z. (2007). Weight gain reduction in mice fed Panax ginseng saponin, a pancreatic lipase inhibitor. J. Agric. Food Chem. 55 (8), 2824–2828. 10.1021/jf0628025 17367157

[B7] Kim J. H. Yi Y. S. Kim M. Y. Cho J. Y. (2017). “Role of ginsenosides, the main active components of Panax ginseng, in inflammatory responses and diseases.” J. Ginseng Res. 41 (4), 435–443. 10.1016/j.jgr.2016.08.004 29021688 PMC5628327

[B8] Kimura K. Sasaki N. Asano A. Mizukami J. Kayahashi S. Kawada T. (2000). “Mutated human beta3-adrenergic receptor (Trp64Arg) lowers the response to beta3-adrenergic agonists in transfected 3T3-L1 preadipocytes.” Horm. Metab. Res. 32 (3), 91–96. 10.1055/s-2007-978597 10786926

[B9] Kumar V. Singh D. D. Lakhawat S. S. Yasmeen N. Pandey A. Singla R. K. (2022). Biogenic phytochemicals modulating obesity: From molecular mechanism to preventive and therapeutic approaches. Evid. Based Complement. Altern. Med. 2022, 6852276. 10.1155/2022/6852276 PMC8977300 35388304

[B10] Lee M. S. Kim C. T. Kim I. H. Kim Y. (2015). “Effects of Korean Red Ginseng extract on hepatic lipid accumulation in HepG2 cells.” Biosci. Biotechnol. Biochem. 79 (5), 816–819. 10.1080/09168451.2014.997186 25774635

[B11] Lee Y. S. Cha B. Y. Yamaguchi K. Choi S. S. Yonezawa T. Teruya T. (2010). “Effects of Korean white ginseng extracts on obesity in high-fat diet-induced obese mice.” Cytotechnology 62 (4), 367–376. 10.1007/s10616-010-9288-7 20862608 PMC2978305

[B12] Li Y. He J. Sui S. Hu X. Zhao Y. Li N. (2012). Clenbuterol upregulates histone demethylase JHDM2a via the β2-adrenoceptor/cAMP/PKA/p-CREB signaling pathway. Cell Signal 24 (12), 2297–2306. 10.1016/j.cellsig.2012.07.010 22820505

[B13] Li Z. Ji G. E. (2018a). “Ginseng and obesity.” J. Ginseng Res. 42 (1), 1–8. 10.1016/j.jgr.2016.12.005 29348715 PMC5766689

[B14] Li Z. Kim H. J. Park M. S. Ji G. E. (2018b). “Effects of fermented ginseng root and ginseng berry on obesity and lipid metabolism in mice fed a high-fat diet.” J. Ginseng Res. 42 (3), 312–319. 10.1016/j.jgr.2017.04.001 29983612 PMC6026359

[B15] Lim S. Park J. Um J. Y. (2019). “Ginsenoside Rb1 induces beta 3 adrenergic receptor-dependent lipolysis and thermogenesis in 3T3-L1 adipocytes and db/db mice.” Front. Pharmacol. 10, 1154. 10.3389/fphar.2019.01154 31680950 PMC6803469

[B16] Liu J. Wang Y. Lin L. (2019). Small molecules for fat combustion: Targeting obesity. Acta Pharm. Sin. B 9 (2), 220–236. 10.1016/j.apsb.2018.09.007 30976490 PMC6438825

[B17] Maggio C. A. Pi-Sunyer F. X. (2003). “Obesity and type 2 diabetes.” Endocrinol. Metab. Clin. North Am. 32 (4), 805–822. 14711063 10.1016/s0889-8529(03)00071-9

[B18] Mitchell B. D. Blangero J. Comuzzie A. G. Almasy L. A. Shuldiner A. R. Silver K. (1998). “A paired sibling analysis of the beta-3 adrenergic receptor and obesity in Mexican Americans.” J. Clin. Invest. 101 (3), 584–587. 10.1172/JCI512 9449691 PMC508601

[B19] Ohyama K. Nogusa Y. Shinoda K. Suzuki K. Bannai M. Kajimura S. (2016). A synergistic antiobesity effect by a combination of capsinoids and cold temperature through promoting beige adipocyte biogenesis. Diabetes 65 (5), 1410–1423. 10.2337/db15-0662 26936964 PMC4839206

[B20] Pietri-Rouxel F. St John Manning B. Gros J. Strosberg A. D. (1997). The biochemical effect of the naturally occurring Trp64-->Arg mutation on human beta3-adrenoceptor activity. Eur. J. Biochem. 247 (3), 1174–1179. 10.1111/j.1432-1033.1997.01174.x 9288945

[B21] Quan H. Y. Yuan H. D. Jung M. S. Ko S. K. Park Y. G. Chung S. H. (2012). “Ginsenoside Re lowers blood glucose and lipid levels via activation of AMP-activated protein kinase in HepG2 cells and high-fat diet fed mice.” Int. J. Mol. Med. 29 (1), 73–80. 10.3892/ijmm.2011.805 21971952

[B22] Ratan Z. A. Haidere M. F. Hong Y. H. Park S. H. Lee J. O. Lee J. (2021). “Pharmacological potential of ginseng and its major component ginsenosides.” J. Ginseng Res. 45 (2), 199–210. 10.1016/j.jgr.2020.02.004 33841000 PMC8020288

[B23] Redman L. M. de Jonge L. Fang X. Gamlin B. Recker D. Greenway F. L. (2007). Lack of an effect of a novel beta3-adrenoceptor agonist, TAK-677, on energy metabolism in obese individuals: A double-blind, placebo-controlled randomized study. J. Clin. Endocrinol. Metab. 92 (2), 527–531. 10.1210/jc.2006-1740 17118998

[B24] Reverte-Salisa L. Sanyal A. Pfeifer A. (2019). “Role of cAMP and cGMP signaling in Brown fat.” Handb. Exp. Pharmacol. 251, 161–182. 10.1007/164_2018_117 29633180

[B25] Riobó Serván P. (2013). “Obesity and diabetes.” nutr hosp 28. Suppl 5, 138–143. 10.3305/nh.2013.28.sup5.6929 24010754

[B26] Sabouri-Rad S. Sabouri-Rad S. Sahebkar A. Tayarani-Najaran Z. (2017). “Ginseng in dermatology: A review.” Curr. Pharm. Des. 23 (11), 1649–1666. 10.2174/1381612822666161021152322 27774902

[B27] Shehadeh M. B. Suaifan G. Abu-Odeh A. M. (2021). “Plants secondary metabolites as blood glucose-lowering molecules.” *Molecules* 26(14). 10.3390/molecules26144333 PMC8307461 34299610

[B28] Sipilainen R. Uusitupa M. Heikkinen S. Rissanen A. Laakso M. (1997). “Polymorphism of the beta3-adrenergic receptor gene affects basal metabolic rate in obese Finns.” Diabetes 46 (1), 77–80. 10.2337/diabetes.46.1.77 8971085

[B29] So S. H. Lee J. W. Kim Y. S. Hyun S. H. Han C. K. (2018). “Red ginseng monograph.” J. Ginseng Res. 42 (4), 549–561. 10.1016/j.jgr.2018.05.002 30337816 PMC6190493

[B30] Wu J. Bostrom P. Sparks L. M. Ye L. Choi J. H. Giang A. H. (2012). “Beige adipocytes are a distinct type of thermogenic fat cell in mouse and human.” Cell 150 (2), 366–376. 10.1016/j.cell.2012.05.016 22796012 PMC3402601

[B31] Wu Y. Yu Y. Szabo A. Han M. Huang X. F. (2014). “Central inflammation and leptin resistance are attenuated by ginsenoside Rb1 treatment in obese mice fed a high-fat diet.” PLoS One 9 (3), e92618. 10.1371/journal.pone.0092618 24675731 PMC3968027

[B32] Yoo D. G. Kim M. C. Park M. K. Song J. M. Quan F. S. Park K. M. (2012). “Protective effect of Korean red ginseng extract on the infections by H1N1 and H3N2 influenza viruses in mice.” J. Med. Food 15 (10), 855–862. 10.1089/jmf.2012.0017 22856395 PMC3466917

[B33] Yu J. S. Roh H. S. Baek K. H. Lee S. Kim S. So H. M. (2018). “Bioactivity-guided isolation of ginsenosides from Korean Red Ginseng with cytotoxic activity against human lung adenocarcinoma cells.” J. Ginseng Res. 42 (4), 562–570. 10.1016/j.jgr.2018.02.004 30337817 PMC6190500

[B34] Zhang L. Virgous C. Si H. (2017). Ginseng and obesity: Observations and understanding in cultured cells, animals and humans. J. Nutr. Biochem. 44, 1–10. 10.1016/j.jnutbio.2016.11.010 27930947

[B35] Zhang Z. Yang D. Xiang J. Zhou J. Cao H. Che Q. (2021). “Non-shivering thermogenesis signalling regulation and potential therapeutic applications of Brown adipose tissue.” Int. J. Biol. Sci. 17 (11), 2853–2870. 10.7150/ijbs.60354 34345212 PMC8326120

[B36] Zhao Y. Chen B. Shen J. Wan L. Zhu Y. Yi T. (2017). “The beneficial effects of quercetin, curcumin, and resveratrol in obesity.” Oxid. Med. Cell Longev. 2017, 1459497. 10.1155/2017/1459497 29138673 PMC5613708

